# Pneumatic Bellow Actuator with Embedded Sensor Using Conductive Carbon Grease

**DOI:** 10.3390/s24165403

**Published:** 2024-08-21

**Authors:** David Moreno, Diana Narvaez, Brittany Newell

**Affiliations:** Polytechnic Institute, School of Engineering Technology, Purdue University, West Lafayette, IN 47907, USA; hmorenor@purdue.edu (D.M.); narvaez@purdue.edu (D.N.)

**Keywords:** dielectric electroactive polymers, soft actuator, conductive carbon grease, pressure sensor, pneumatic actuator

## Abstract

The present work demonstrates the manufacturing process of a pneumatic bellow actuator with an embedded sensor, utilizing a novel manufacturing approach through the complete use of additive manufacturing techniques, such as direct ink writing (DIW) and traditional fused deposition modeling (FDM) methods. This study is innovative in its integration of a dielectric electroactive polymer (DEAP) structure with sensing electrodes made of conductive carbon grease (CCG), showcasing a unique application of a 3D-printed DEAP with CCG electrodes for combined DEAP sensing and pneumatic actuation. Initial experiments, supported by computational simulations, evaluated the distinct functionality of the DEAP sensor by itself under various pressure conditions. The findings revealed a significant change in capacitance with applied pressure, validating the sensor’s performance. After sensor validation, an additive manufacturing process for embedding the DEAP structure into a soft pneumatic actuator was created, exhibiting the system’s capability for dual sensing and actuation, as the embedded sensor effectively responded to applied actuation pressure. This dual functionality represents an advancement in soft actuators, especially in applications that require integrated and responsive actuation and sensing capabilities. This work also represents a preliminary step in the development of a 3D-printed dual-modality actuator (pneumatic and electrically activated DEAP) with embedded sensing.

## 1. Introduction

The evolution of smart materials and additive manufacturing technologies has opened new possibilities for designing and developing flexible actuators and sensors [1,2]. These devices have potential applications in various fields, including robotics, automotive, medicine, and consumer electronics [1,2]. One of the critical challenges in the design of flexible actuators is the integration of sensing systems that do not compromise the flexibility and functionality of the device [3,4]. Dielectric electroactive polymers (DEAPs) are materials that have shown promise in this regard due to their ability to change shape and size in response to an applied electric field [3,4,5,6]. DEAPs, consisting of a dielectric material placed between two conductive electrodes, possess a dual capability; DEAPs can generate an electrical signal from a mechanical input while also generating significant deformation when exposed to an electric field [3,4,5,7]. This distinctive property makes them ideal for sensing applications, where changes in the device’s capacitance can be used to measure compressive forces, as well as actuation [3,4,5,8].

A key focus of this study is the development of a pneumatic actuator with embedded sensors that integrate both sensing and actuation capabilities. This work leverages innovative additive manufacturing techniques such as direct ink writing (DIW) and Fused Deposition Modeling (FDM). These methods provide precise control over material deposition, facilitating the integration of both pneumatic and electrically activated DEAP sensors and actuator systems. The combination of DIW and FDM represents a novel approach for creating actuators capable of dual activation mechanisms, enhancing the versatility and functionality of these devices in various applications, including soft robotics and biomedical devices [2,4,5,9].

The field of 3D-printed pneumatic actuators has grown, as demonstrated by Drotman et al. [10]. In their work, they explored the development of 3D-printed, three-chambered pneumatic actuators with bellows, designed to achieve controlled motion through pressure modulation. This study highlighted the advantages of 3D printing for rapid prototyping and the flexibility of soft robotics applications. Additionally, Ahn et al. [11] specifically focused on the development of thermopneumatic soft micro bellow actuators, utilizing internal heating mechanisms for precise actuation control, while Xavier et al. [12] emphasized the potential of these actuators in delicate manipulation tasks. Reis Carneiro et al. [13] explored biphasic Ag-EGaIn electrodes to enhance electromechanical properties and energy efficiency. Bernat et al. [14] demonstrated that incorporating silicone-based membranes in DEAPs can improve their flexibility and response time. These advancements underscore the potential of such materials for developing advanced actuators and sensors, as also reviewed by Kim and Tadokoro [15] in their overview of electroactive polymers for robotic applications. Furthermore, Zhou et al. [16] discussed integrated actuation and sensing strategies for soft robots, emphasizing the importance of combining these functions to achieve intelligence and autonomy. This integrated approach is critical for advancing the capabilities of soft robots, enabling them to interact more effectively with their environments and perform complex tasks.

This study presents an example of a fully additively manufactured dielectric electroactive polymer actuator using direct ink writing technology. Initially, a DEAP sensor was developed using conductive carbon grease (CCG) embedded in a dielectric matrix composed of thermoplastic polyurethane (TPU). This first prototype served as a foundational experiment to evaluate the sensor’s functionality and to optimize the manufacturing process for these two materials. Building on this, a second prototype was created that integrates the DEAP sensor with a pneumatic actuator, utilizing the same materials and additive manufacturing techniques. Both prototypes were systematically tested through experimental trials and computational simulations to evaluate their performance under various pressure conditions. The subsequent sections detail the fabrication process, the experimental setup, and the results of these tests, providing insights into the capabilities of these actuators and their potential applications in flexible electronics, robotics, and biomedical devices [17,18,19,20]. This work aims to contribute to the ongoing development of high-performance, flexible actuators and sensors, offering a foundation for future innovations in this field.

## 2. Literature Review and Foundational Concepts

### 2.1. Pneumatic Actuators

Pneumatic actuators are a versatile category of soft actuators that offer significant flexibility and adaptability. These actuators operate by pressurizing air chambers within a soft, often elastomeric structure, causing the actuator to expand, contract, or bend. The design allows for a greater range of motion compared to rigid structures, enabling applications in soft robotics, medical devices, and industrial automation. As demonstrated by Drotman et al. [10], the geometric parameters of the pneumatic bellows, such as wall thickness and chamber dimensions, critically influence the actuator’s performance, including bending angles and blocked force.

### 2.2. Electroactive Polymers (EAPs)

Research on electroactive polymers (EAPs) has grown significantly due to their unique properties and potential in various applications, such as actuators, sensors, and power generation devices [3,4,21,22,23]. Among these, dielectric electroactive polymers (DEAPs) stand out because they can produce substantial deformations while maintaining low stiffness, making them ideal for applications that require both flexibility and high sensitivity [9,21].

EAPs are mainly divided into two categories: ionic EAPs and electrical EAPs. Ionic materials include metal ionic polymers and polyelectrolyte gel, while electrical materials include dielectric elastomers and ferroelectric polymers [3,4,24]. Electrical EAPs in particular have shown considerable promise for developing adaptable actuators and sensors, as they can generate large deformations with relatively low voltages [1,3,9].

### 2.3. Dielectric Electroactive Polymers (DEAPs)

DEAPs are electrical EAPs composed of a dielectric layer located between two conductive electrodes. This structure allows the material to undergo significant deformation when an electric field is applied, changing its capacitance [25,26,27]. DEAPs are used in various applications, including pressure sensors, power generators, and artificial muscles [1,14,27,28,29].

The fundamental equation that describes the behavior of DEAPs is Maxwell’s equation, which states that the capacitance *C* of a capacitor is given by

(1) $$\;\;\;\;\;\;\;C=\frac{\epsilon_{0}\epsilon_{r}A}{d}$$

where

*C* is the capacitance in Farads;

$\epsilon_{0}$ is the permittivity of free space;

$\epsilon_{r}$ is the dielectric constant of the material;

*A* is the area of the electrodes;

*d* is the distance between the electrodes.

This equation (Equation (1)) shows the relationship between the physical properties of the dielectric material and its capacitance [30].

### 2.4. Integration of Sensors in Flexible Actuators

Conventional sensors often have limitations in terms of flexibility, which can restrict the functionality of actuators [9,31]. Various strategies have been explored to address this problem, including using composite materials and incorporating sensing elements directly into the actuator structure [24,26,32,33]. Combining DEAPs with integrated sensors allows for the creation of devices that can measure compressive forces and changes in capacitance, providing accurate data on environmental and operational conditions [24,32]. An example of this integration is the development of flexible pressure sensors that use dielectric polymers [1]. These sensors can measure the applied pressure by detecting changes in the material’s capacitance when it is deformed. The formula that relates the capacitance to the applied pressure can be expressed as

(2) $$\;\;\;\;\;\;\;\;C\left(P\right)=\frac{\epsilon_{0}\epsilon_{r}A}{d-\Delta d\left(P\right)}$$

where

*P* is the applied pressure;

$\Delta d\left(P\right)$ is the change in the distance between the electrodes due to the pressure.

This equation (Equation (2)) shows that as the pressure increases, the distance between the electrodes decreases, increasing capacitance [26].

### 2.5. Additive Manufacturing and Flexible Sensors

Additive manufacturing has become a powerful technique for creating devices with complex geometries and customized properties [10,30,31]. In the context of DEAPs, 3D printing enables rapid and low-cost manufacturing of customized sensors and actuators [10,11,24]. Previous studies have demonstrated the feasibility of using additive manufacturing technologies to design DEAPs, including work by Gonzalez et al., who developed flexible sensors using 3D-printed dielectric polymers [2,5]. Another study by Fu et al. also highlights the use of 3D printing for creating complex DEAP structures [3,19,21].

The 3D printing of DEAPs not only facilitates the creation of complex structures but also allows for the integration of conductive and dielectric materials in a single manufacturing process, thus improving the devices’ efficiency and performance [24,31]. Advanced materials such as thermoplastic polyurethane (TPU), known for its stretchability and durability, are ideal for applications in flexible actuators [10].

### 2.6. Carbon Conductive Grease (CCG) in Actuators and Sensors

Carbon conductive grease (CCG) is a composite material that combines a grease matrix’s flexibility with carbon’s high conductivity. This allows for the creation of adaptable electrodes that can be easily integrated into dielectric polymer structures [33,34,35].

The inclusion of CCG in 3D-printed devices has shown significant improvements in the performance of flexible actuators and sensors. Researchers have shown that adding CCG to dielectric polymer structures improves the actuators’ sensing capability and efficiency, allowing for faster and more accurate responses to applied forces [36].

A key aspect of using CCG is its ability to maintain electrical conductivity under significant deformations, which is crucial for applications in sensors that must operate under dynamic conditions [13,14,23]. The formula that describes the relationship between the conductivity of the grease and the applied deformation is

(3) $$\;\;\;\;\;\;\;\;\sigma =\sigma_{0}\left(1-\frac{\Delta L}{L}\right)$$

where

σ is the conductivity under deformation.

$\sigma_{0}$ is the initial conductivity.

ΔL is the change in the length of the material due to deformation.

L is the initial length of the material.

This relationship (Equation (3)) shows how the conductivity of the CCG is reduced when the material is stretched. However, since TPU is a viscoelastic material, it can withstand large deformation [20].

Within the scope of this research, the actuator system presented in this work demonstrates a synergistic integration of dielectric electroactive polymers (DEAPs), carbon conductive grease (CCG), and pneumatic actuation.

## 3. Materials and Methods

### 3.1. Material Selection

Material selection was critical for successful prototype development. Thermoplastic polyurethane (TPU) specifically Filaflex 60A [37] was chosen as the base material for the dielectric in the DEAP sensor and the base material for the pneumatic actuator due to its electrical and mechanical properties as well as 3D-printability. Its hyperelastic nature provides high stretchability and flexibility for pneumatic actuation. Additionally, TPU is highly durable and resistant to abrasion, enabling it to endure repetitive mechanical stresses without significant degradation, which makes it ideal for dynamic applications such as actuation mechanisms. In regard to DEAP sensing, TPU has a high dielectric constant, approximately 6.32, making it ideal for pressure monitoring through capacitance-based sensing [5].

For the electrode material, carbon conductive grease (CCG) [38] was selected. CCG consists of carbon black particles suspended in a silicone oil matrix, offering a combination of high conductivity and mechanical compliance. This allows CCG to function effectively as an electrode material in DEAPs, accommodating significant deformation without compromising electrical performance. Traditionally, the electrode layer in DEAP sensors and actuators is stiffer than the dielectric layer due to the addition of conductive particles within a hyperelastic polymer matrix. This stiffness limits actuation and sensing potential and can cause separation of these layers during motion. By incorporating grease as the electrode, issues of layer separation and movement restriction are mitigated. CCG was also chosen because it has been demonstrated to work effectively as a conductive electrode in a 3D-printed DEAP actuator with TPU as the dielectric layer, as shown by Gonzalez et al. [2,5].

Previous studies have also demonstrated the benefits of combining TPU and CCG in DEAP actuators. Gonzalez et al. [5] presented a novel design of a DEAP actuator showcasing the successful integration of TPU and CCG. Their work highlighted significant improvements in durability and responsiveness, allowing for high actuation strains while minimizing energy loss due to mechanical resistance. The work in this study focuses on the development of a fully additive method to produce pneumatic structures with CCG-based DEAP sensors, while also serving as a preliminary step for future incorporation of dual actuation modes, including pneumatic and traditional high-voltage DEAP actuation.

### 3.2. Manufacturing Process

#### 3.2.1. Proof of Concept (Prototype 1 DEAP Sensor)

**Figure 1:**
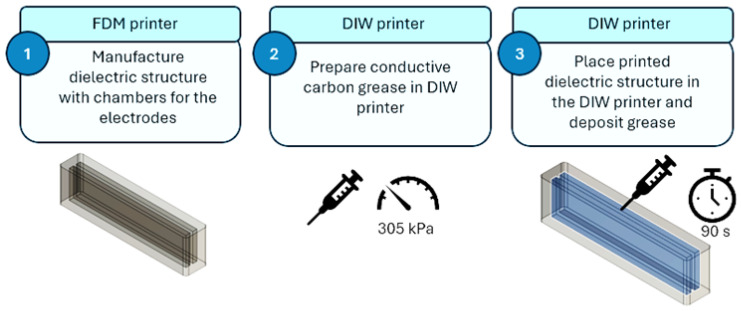
Flowchart for manufacturing of Prototype 1.

An initial prototype of a DEAP sensor utilizing conductive carbon grease was developed (Figure 1), drawing inspiration from previous work by Rodriguez et al. [2,5]. This prototype served as a proof of concept and a foundation for optimizing the manufacturing process for integrating conductive carbon grease. The aim was to validate the feasibility of the design and to identify areas for refinement in the material deposition and integration techniques.

**Figure 2:**
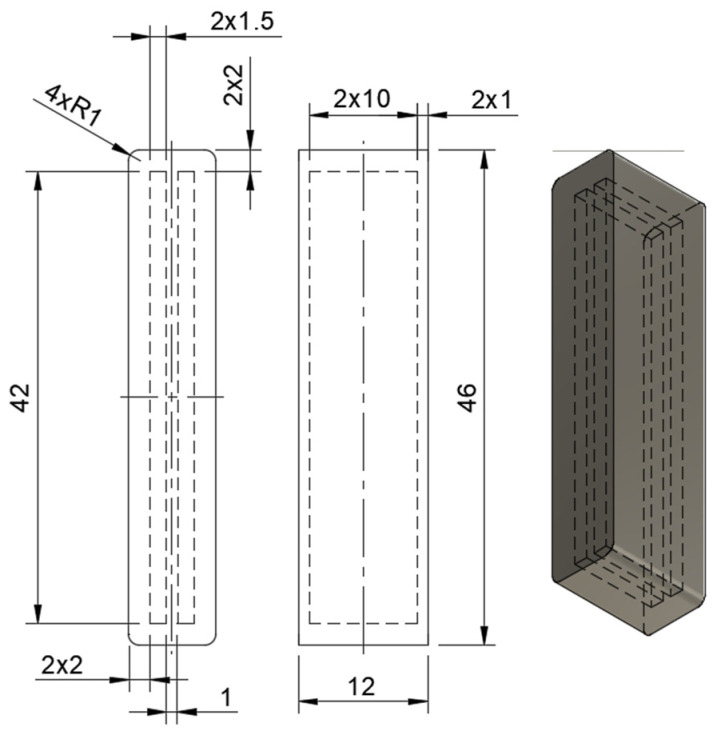
Design of capacitor structure made of flexible TPU and two chambers of conductive carbon grease.

The structure is composed of a dielectric matrix made of thermoplastic polyurethane, TPU 60A by Filaflex [37], and includes two inner chambers filled with conductive carbon grease (CCG). These grease-filled chambers serve as the electrodes of a parallel plate capacitor, forming a DEAP sensor. Figure 2 shows the design of Prototype 1, where the units are in mm.

#### 3.2.2. Manufacture of Dielectric Structure (Prototype 1)

The dielectric structure was printed using traditional FDM techniques on a Lulzbot TAZ 6 printer [39]. The print parameters were experimentally optimized to achieve a high-quality print surface while minimizing air leakage in the pneumatic actuator. Specifically, the chamber size and layer height were adjusted for optimal performance. The inner chambers, as shown in Figure 2, maintain the same length and width as the design presented by Rodriguez et al. [13], ensuring both sensor functionality and printability:

- Layer height: 0.25 mm;
- Infill density: 100%;
- TPU printing temperature: 215 °C;
- Build plate temperature: 70 °C;
- Printing speed: 60 mm/s.

#### 3.2.3. Deposition of Conductive Grease into Dielectric Structure (Prototype 1)

Deposition of conductive carbon grease was performed using DIW on a Cellink Inkredible+ printer [40]. As the deposition was performed at specific points on the dielectric structure, the position of the nozzle was static and a pressure of 305 kPa was set to ensure continuous flow of the conductive grease. A 20-gauge nozzle syringe with an external diameter of 0.91 mm and a length of 12.7 mm was selected based on conductive grease viscosity (80.4 Pa*s) [41] and approximate particle size (~100 µm). Experimentation with nozzle sizes and pressures showed that the 20-gauge nozzle and 305 kPa of applied extrusion pressure were optimal for continuous flow of grease [42].

The thickness of the structure was optimized so that the syringe used for grease deposition was a compression fit in the chambers to maximize fill and minimize over-extrusion. A height of 10 mm was set based on the length of the needle. The side walls were 2 mm thick, while the top and bottom walls were set to 1 mm. The top/bottom thickness of 0.8 mm was defined to prevent the conductive grease from leaking out of the chambers after being penetrated by the needle.

**Figure 3:**
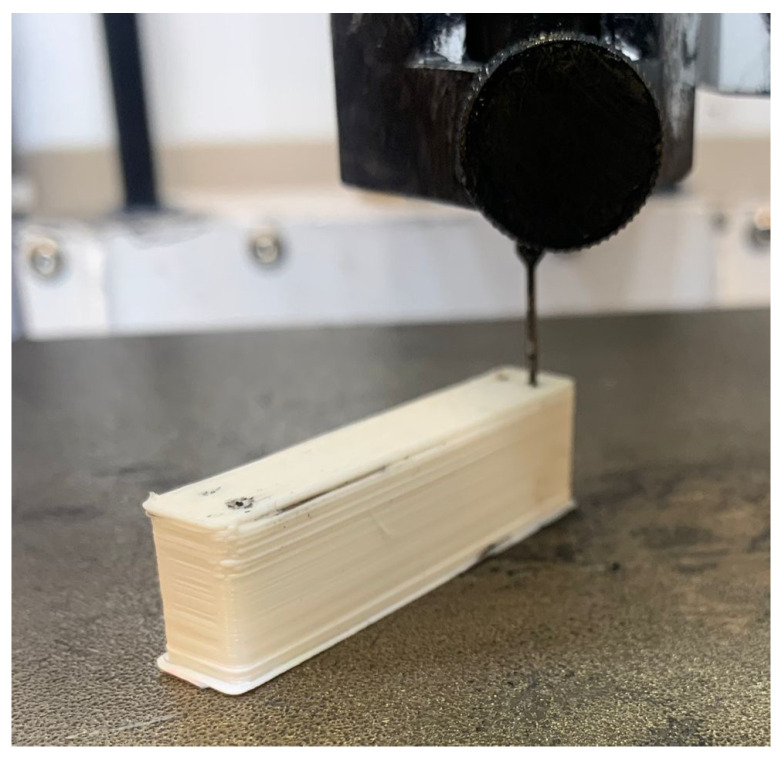
CellInk Bioprinter pressurized syringe injection of conductive grease into the chambers of the Prototype 1 DEAP sensor.

Two additional layers at the top contributed to fully sealing the chambers. The print was paused before making the last two layers/top cover. While paused, the print was taken to the DIW printer and grease was injected into each of the chambers through two holes at each chamber. For each hole, the grease flowed for 90 s at a pressure of 305 kPa. Figure 3 shows the process of injecting carbon grease into a chamber of the sensor prototype. After the addition of grease, the print was resumed on the FDM Lulzbot Taz 6 printer.

#### 3.2.4. Manufacture of Prototype 2 (DEAP Sensor + Pneumatic Actuator)

**Figure 4:**
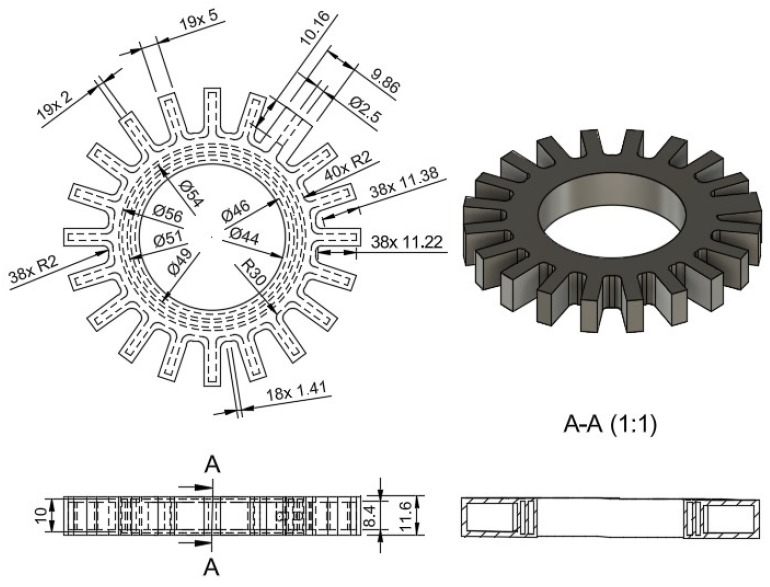
Dimensions of Prototype 2. Units in mm.

For the design of the actuator with an embedded DEAP sensor, a circular pneumatic actuator shape was chosen to apply the pressure uniformly throughout the cylindrical object. The walls surrounding the air chamber were optimized through experimental tests so that the walls could withstand the pressure with the lowest thickness. Similarly, the walls surrounding the chambers of conductive grease were optimized so that they could prevent conductive carbon grease from leaking with the lowest thickness. Figure 4 shows the dimensions of Prototype 2.

The height of the air chamber is lower than the height of the chambers for electrodes with values of 8.4 mm and 10 mm, respectively. This ensures that the air chamber is sealed on top while the CGG is deposited in the chambers. The height of Prototype 2 is 11.6 mm, and the diameter of the air inlet is 2.5 mm.

#### 3.2.5. Manufacture of Dielectric Structure (Prototype 2)

For Prototype 2, the manufacturing process closely followed the procedure used for Prototype 1. The printing parameters remained the same, with the exception of the infill pattern. A linear infill pattern was chosen for Prototype 2 to achieve a smoother surface, in contrast to the zigzag pattern utilized in Prototype 1.

#### 3.2.6. Deposition of Conductive Grease into Dielectric Structure (Prototype 2)

**Figure 5:**
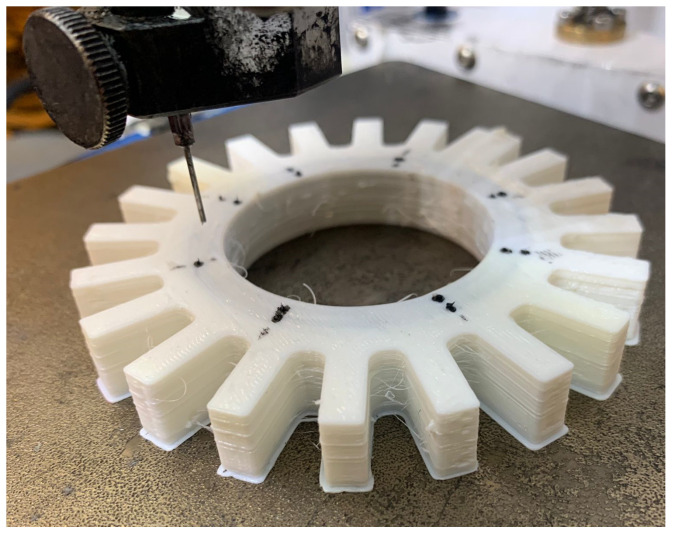
CellInk Bioprinter pressurized syringe injection of carbon conductive grease into the chambers of Prototype 2.

The print was paused before the top two layers were made and the actuator was placed on the bioprinter for the injection of conductive grease. In this case, eight injection points were defined, and the grease flowed for 90 s for each point at a pressure of 305 kPa. Subsequently, the two remaining layers of the print covered the holes. Figure 5 shows the process of injecting the grease in Prototype 2.

### 3.3. Computational Simulations

#### 3.3.1. Computational Simulations on Prototype 1

An experimentally validated finite element analysis model was constructed to predict the working pressure range and expected actuator deformation for the prototype. After experimental validation, the model was intended to be used for testing alternate geometries. The finite element model for Prototype 1 was built in COMSOL Multiphysics 5.5 software. COMSOL was selected due to its ability to integrate multiple physics domains, in this case electrostatics and traditional mechanical analysis, along with tools for accurate calculation of the deformation of hyperelastic materials. Once experimentally validated, the model was used to test the impact of variables on the actuator to optimize design and experimentation. Key functionalities utilized in COMSOL for this design were the use of moving mesh methods and alternative solver configurations such as iterative, direct, segregated, or fully coupled to improve accuracy and processing efficiency. The mesh of the structure was generated by the software based on the physics involved defined as physics-controlled meshing with an element size denominated as “finer”, obtaining a total of 9587 elements including triangle prisms and tetrahedrals.

To input the properties of the material into the model, a previous characterization of the material was referenced. In this work, a tensile test according to the ASTM D638-14 standard [43] was conducted on 3D-printed TPU and a least square curve fit was obtained with the data of stress versus strain using ANSYS 2022 [44]. The values for $C_{10}$ and $C_{01}$ were estimated as 0.28 and 0.59 MPa, respectively.

**Figure 6:**
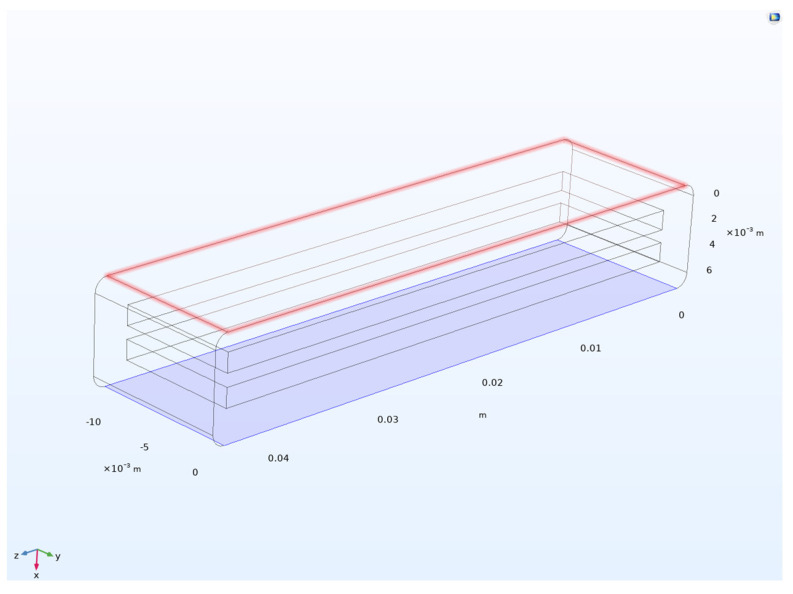
Boundary load on top and fixed constraint at the bottom for Prototype 1.

The geometry of the structure was imported directly from the 3D-printer STL file. Then, each of the physics for the model were added, including solid mechanics, electrostatics, and electromechanics. As per the solid mechanics module, a fixed constraint and a boundary load were set at the bottom and top of the structure (blue and red highlighted in Figure 6). The boundary load was in the form of a sweep ranging from 0 to 60 psi (0 to 413.69 kPa) with a step of 1 psi (6.89 kPa). The sweep function enabled convergence before movement to the next step due to high deformation, which results from the hyperelastic behavior of the material.

**Figure 7:**
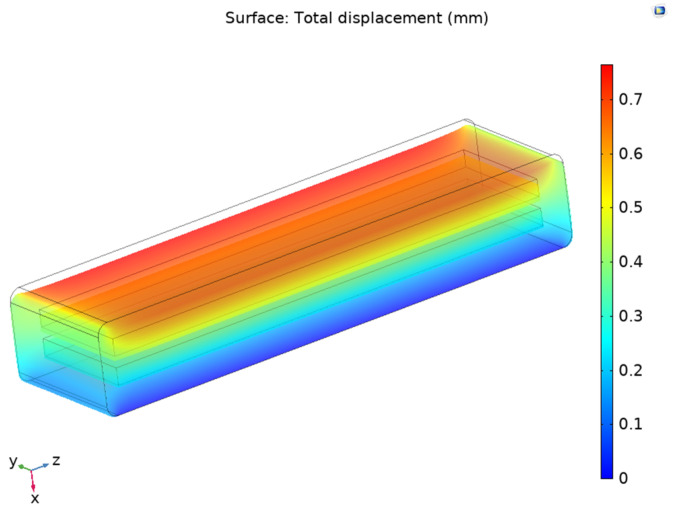
Total displacement of Prototype 1.

As per the electrostatic module, the boundaries of one of the electrodes was defined as the ground while the boundaries of the remaining electrode were defined as the terminal with a voltage of 1V. The computational simulation used quadratic elements and implicit analysis. After the finite element model was computed, the structure experienced a deformation, as shown in Figure 7. The highest deformation occurred on the face where the pressure was applied, while there was minimal deformation on the face of the fixed constraint. The maximum value for displacement was 0.567 mm.

The deformation produced a reduction in the thickness of the dielectric structure, leading to a reduction in the distance between the electrodes. Such reduction leads to an increase in the capacitance of the structure, as seen in Equation (1).

**Figure 8:**
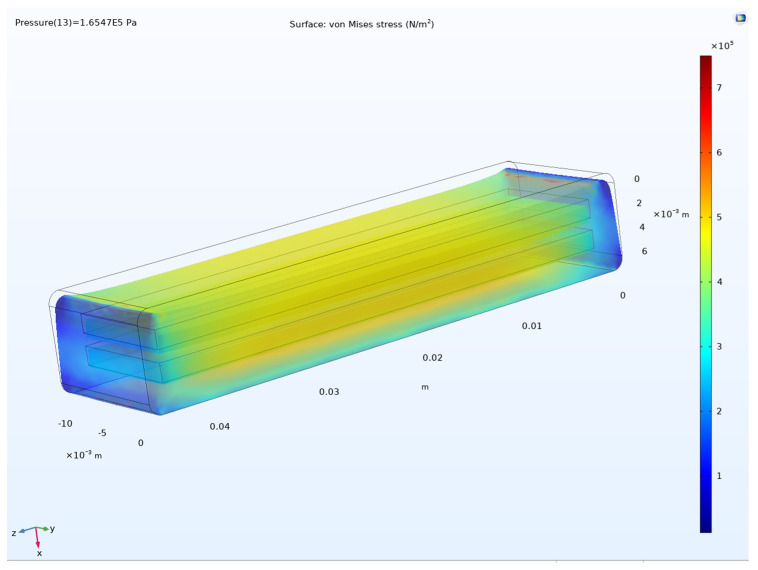
Von Mises stress in the DEAP sensor Prototype 1.

Figure 8 shows the von Mises stress of Prototype 1. The stress in the structure was below 1MPa, which is considered a low-stress condition. The highest stress occurred in the face of the fixed constraint.

**Figure 9:**
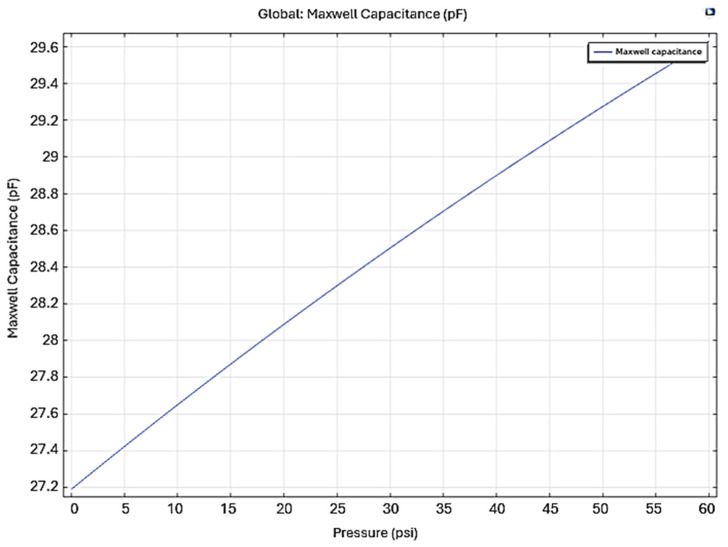
Maxwell capacitance as function of the pressure applied to Prototype 1.

The deformation produces an increase in capacitance, and the Maxwell capacitance versus the applied pressure is shown in Figure 9. The increase in capacitance corresponds to an increase in the applied pressure, which demonstrates the functionality of the structure as a sensor.

#### 3.3.2. Computational Simulations for Prototype 2

**Figure 10:**
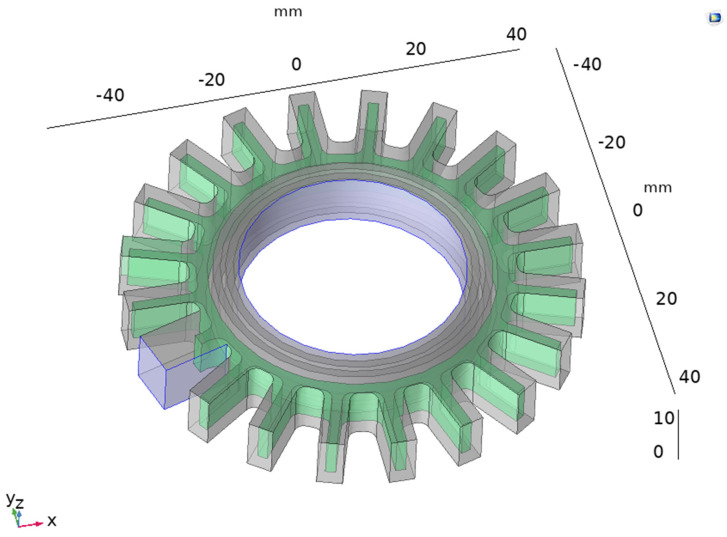
Boundary load applied to the inner channel of the actuator (in blue) and fixed boundary condition applied to the face in contact with the object and two perpendicular faces near the inlet of Prototype 2.

The computational model for Prototype 2 was built using the same material and parameters as those used for Prototype 1. However, the geometry of the actuator was more complex; the meshing required a higher number of elements in the mesh and the step of the sweep of pressures was reduced to 0.1 psi (0.689 kPa). In this design, pressure was applied to the inner channel of the actuator and fixed constraints were added at the inlet of the actuator and the face in contact with the object, as shown in Figure 10.

**Figure 11:**
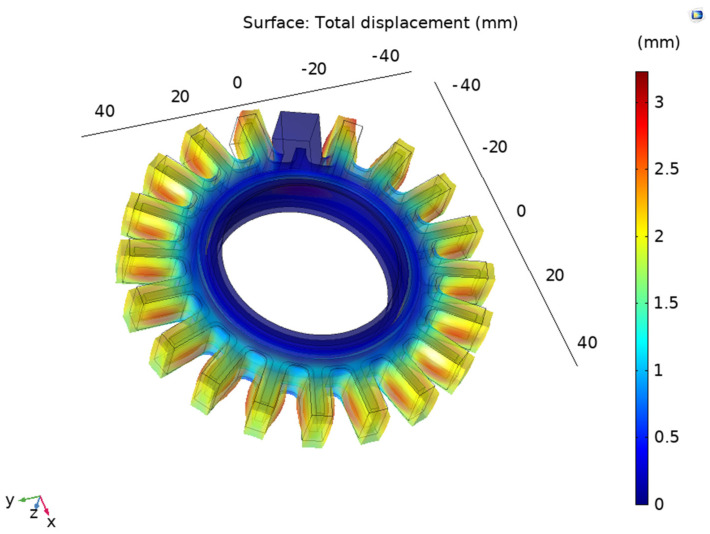
Total displacement of Prototype 2.

The model converged after 201 iterations, using a step size of 0.1 psi (0.689 kPa), and the maximum value of pressure was 30 psi. The mesh contained 685,028 elements. As per the displacement, the highest value was observed in the outside bellows and in the portion between the electrodes (as desired). The total displacement is shown in Figure 11.

**Figure 12:**
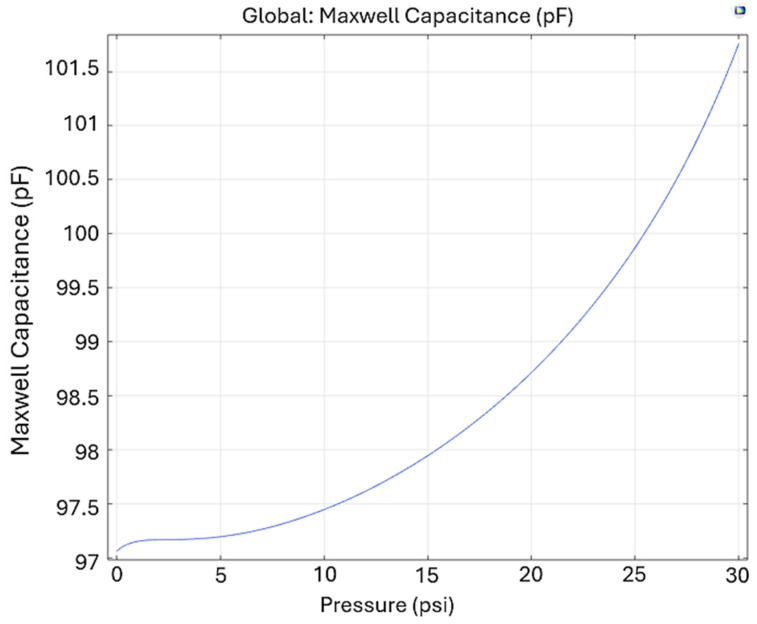
Maxwell capacitance as a function of the pressure applied to Prototype 2.

The Maxwell capacitance as a function of the applied pressure is shown in Figure 12. Given the hyperelastic nature of the TPU, a nonlinear behavior is observed as the deformation directly affects the value of capacitance. It is observed that at 30 psi (206.84 kPa), the value of Maxwell capacitance is 101.7 pF, which corresponds to an increase of 5.15% with respect to the initial value of capacitance at 0 psi.

**Figure 13:**
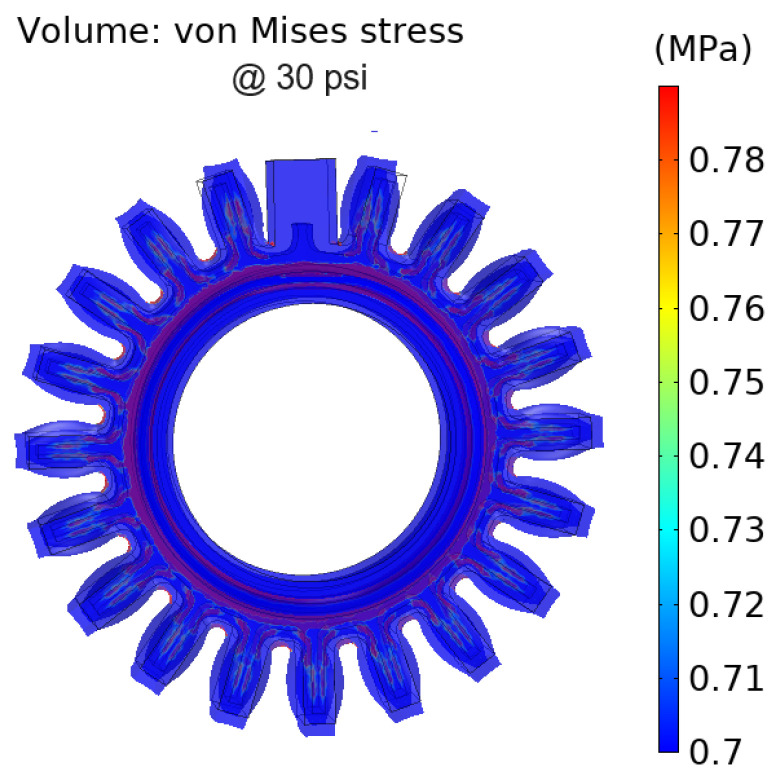
Von Mises stress of Prototype 2.

Figure 13 shows the von Mises stress of the structure. The maximum stress is below 1 MPa, corresponding to a low-stress condition. For better visualization, stresses below 0.78 MPa are shown in Figure 13.

### 3.4. Experimental Tests

**Figure 14:**
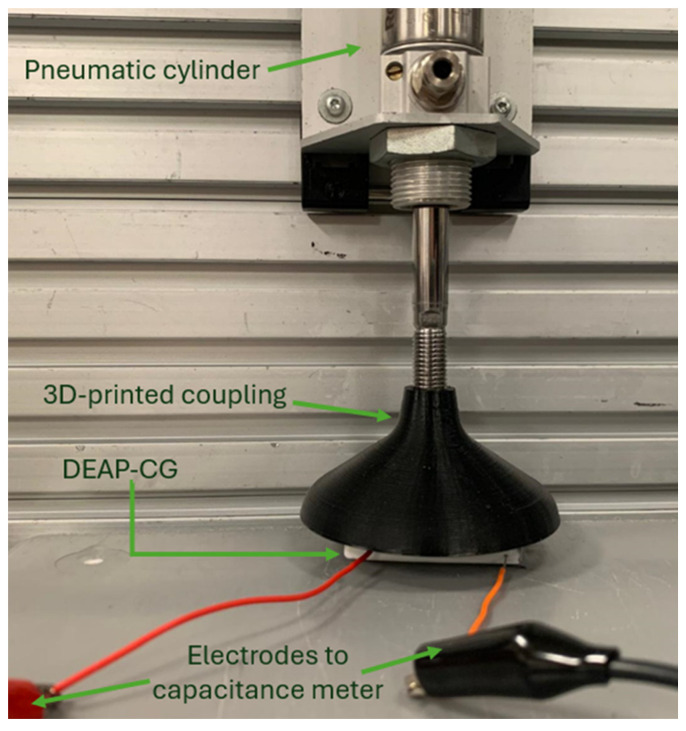
Setup for pressure testing on Prototype 1.

Once the conductive grease was deposited into the channels of Prototype 1, leads were connected to each of the chambers by pushing 28 AWG wires against the top face of the sensor until they penetrated the flexible material and contacted the conductive grease. Initial measurements of capacitance were performed using an LCR meter, model 879B [45]. The 879B model allows USB communication to facilitate data gathering on a laptop. A pneumatic system was set up to perform pressure tests on the sensor. The setup consisted of a pneumatic cylinder, model 0-822-334-510, manufactured by Bosch Rexroth [8]. The pneumatic cylinder withstands pressures up to 145 psi (999.74 kPa). To apply uniform pressure on one side of Prototype 1, a 3D-printed coupling was designed (shown in black in Figure 14). The pressure was set between 0 and 60 psi (413.69 kPa) with increments of 1 psi during the first 10 psi and 10 psi (68.95 kPa) steps afterwards. For each pressure condition, the capacitance was recorded for 30 s, repeating the process for five samples with five repetitions each. The setup for the pressure test on Prototype 1 is shown in Figure 14.

**Figure 15:**
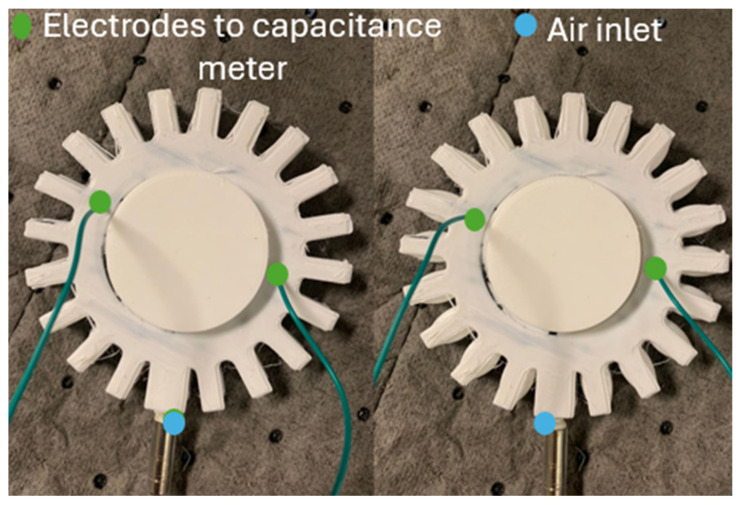
Experimental test for Prototype 2 at 0 (**left**) and 30 psi (206.84 kPa) (**right**).

Regarding Prototype 2, preliminary tests were performed to identify the maximum pressure the actuator was capable of withstanding before the conductive carbon grease started to leak. The pressure was set from 0 to 10 psi (0 to 68.95 kPa) with a step of 1 (6.89 kPa) psi and then to 30 (206.84 kPa) psi with a step of 5 psi (34.4738 kPa), repeating the process for five samples with five repetitions each. Figure 15 presents the setup for the experimental test on Prototype 2. A smaller step size, 1 psi, was used for 0–10 psi because of the nonlinear behavior seen during the first 10 psi applied, which is thought to be due to grease redistribution into remanent voids during initial pressurization. After 10 psi, the nonlinearity decreases, and a larger step size can be used. This finding is consistent with past work. Gonzalez et al. [2] measured capacitance at small intervals during the first 10 psi applied on a similar DEAP device, to track the charging capacitor behavior at this initial range.

## 4. Results

**Figure 16:**
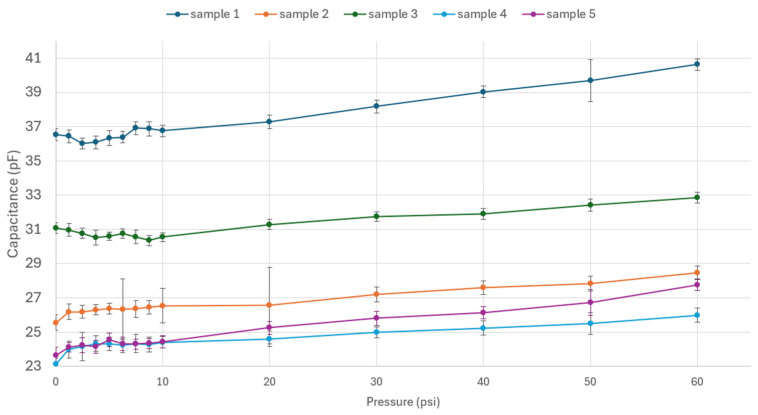
Capacitance vs. pressure for Prototype 1.

Figure 16 shows the results of the experiments conducted on Prototype 1, focusing on the sensor structure. These results show an increase in capacitance as the pressure increases for all five samples. For samples 2, 4, and 5, the pressure increases notably from rest to 1 psi (6.89 kPa), followed by an increasing behavior. For samples 1 and 3, no clear trend was observed for the first 10 psi (68.95 kPa), which might be attributed to the uneven distribution of the pressure over one face of the structure, given that the chambers were filled with extra carbon grease, generating expansion on the entire structure. An excess of carbon grease can also explain the higher ranges in capacitance for samples 1 and 3.

It can be observed that the experimental results from samples 2, 4, and 5 align with the expected ranges from the COMSOL simulation. Also, for both the computational simulation and the experimental results, there is an increase in capacitance in response to the input pressure. Given that each sample has a different value for initial capacitance, the normalized capacitance was calculated using the following equation:

(4) $$\frac{\Delta C}{C_{0}}=\frac{C-C_{0}}{C}\;$$

**Figure 17:**
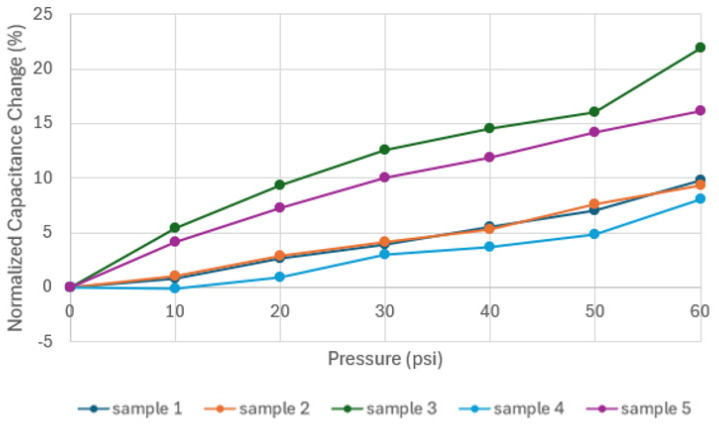
Normalized capacitances change vs. pressure for Prototype 1.

Normalizing the capacitance change allows for a direct comparison among different samples, given that the value of capacitance in the initial condition varies, as observed in Figure 16. The values of normalized capacitance as a function of the applied pressure for Prototype 1 are shown in Figure 17. For all the samples, an increase is observed between 7 and 23% of their initial capacitance value. These percentages are lower compared to those obtained in a previous study [2,5], where three types of conductive TPU were used as electrodes, with initial capacitance changes between 71 and 141%.

**Figure 18:**
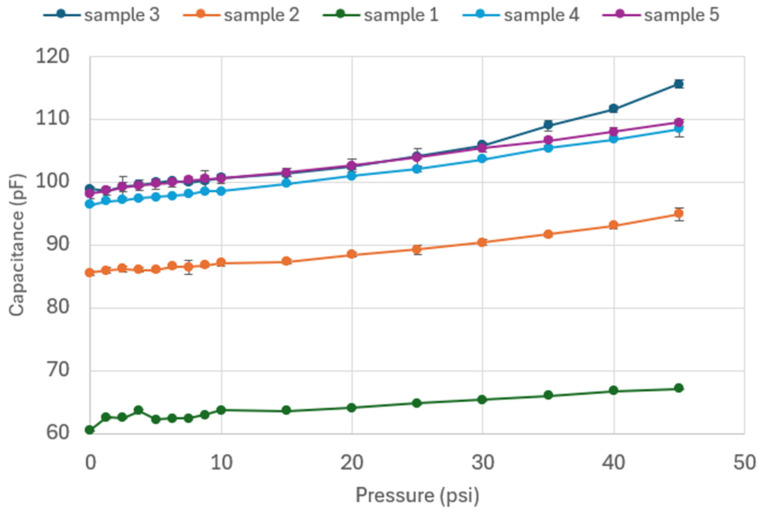
Capacitance vs. pressure for Prototype 2.

The results for the experimental test on Prototype 2 are shown in Figure 18. For all samples, there is an increase in capacitance as the pressure applied to the actuator increases. Different ranges of capacitance among the samples are due to fluctuations in the flow of grease during the injection process. Sensors should be calibrated for normalization of the results prior to use. The fluctuations of grease are a consequence of nozzle clogging due to a build-up of conductive particles. Further research addressing this problem would involve the design of a custom carbon grease, which would balance conductivity and printability with particle size, volume fraction, and purity [34]. The approach of this study focuses on using standard commercial carbon grease, adjusting pressure on the grease and nozzle size, and acknowledging the difference in fill among the samples as a result of particle agglomeration during printing.

**Figure 19:**
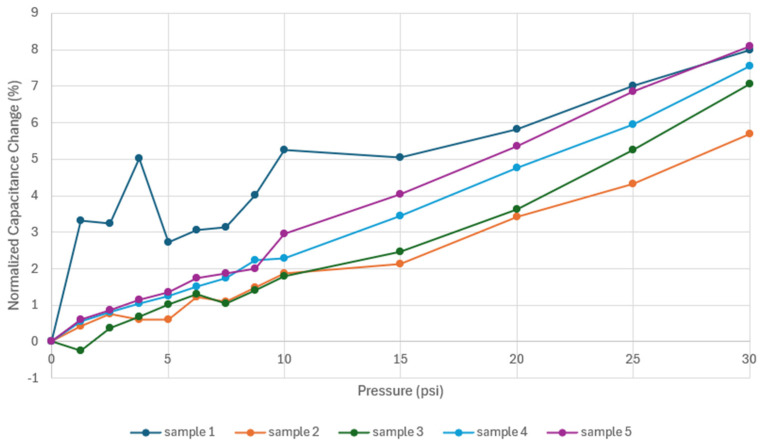
Normalized capacitances change vs. pressure for Prototype 2.

The normalized capacitance change was calculated and computed. The results against applied pressure are shown in Figure 19. It was observed that all samples experienced an increase between 5.5 and 8.5% of their initial value of capacitance. The increase in capacitance in the computational simulation was reported as 5.15%, which is slightly lower than that of the experimental test.

## 5. Discussion

The initial value of capacitance for the five Prototype 1 samples and the five Prototype 2 samples varied with grease distribution in the chambers. However, by computing normalized capacitance change, it was possible to compare the percentage of change among samples. Although the samples showed an increase in initial capacitance, some were more sensitive to applied pressure.

The response of the DEAP sensor, Prototype 1, showed that during the application of 10 psi (68.95 kPa) of pressure, there was no clear trend in the capacitance as a function of the applied pressure. This behavior could indicate an uneven distribution of the carbon grease within the chambers of the electrodes, which may initially be caused by variations in the manufacturing process, particularly in how the carbon grease is deposited. As the pressure increases, the carbon grease redistributes more uniformly, stabilizing the capacitance readings. As observed in Figure 15, the normalized capacitance changes for the five samples ranged between 7% and 23%, allowing for detection of changes in pressure. These results corresponded with those obtained in the computational simulation, where increased capacitance was observed as the pressure increased. The initial prototype experiment served as a baseline for demonstrating the functionality of 3D-printed DEAP sensors using conductive carbon grease. The alignment of experimental results with computational simulations validates the model used and confirms that the observed trends are intrinsic properties of the system. This congruence suggests that with improved uniformity in the distribution of carbon grease, even more consistent sensor performance could be achieved.

Conversely, the subsequent experiments conducted on Prototype 2 demonstrated a clear trend of increasing capacitance as pressure was applied. The geometry of the actuator and the method of applying pressure are crucial for the sensor’s functionality, as pressure should be uniformly distributed throughout the DEAP structure. This consistency in response highlights the effectiveness of the Prototype 2 design and suggests that similar approaches could be adopted to optimize the Prototype 1 system. Ensuring a uniform application of pressure remains a critical factor for reliable sensor outputs.

## 6. Conclusions

These results can be leveraged to further investigate the development of devices incorporating sensors based on DEAPs with conductive carbon grease. These findings highlight the potential of using conductive carbon grease in DEAPs, benefiting from its functionality and minimal impact on the structure’s flexibility. Future research should explore alternative methods of integrating CCG, potentially enhancing the uniformity and repeatability of the sensor’s response. The minimal impact of CCG on the flexibility of the DEAP structure also opens possibilities for more versatile applications, particularly in fields requiring highly adaptable sensors.

In terms of actuation, DEAP structures respond mechanically to applied electrical input (high voltage, low current), which means they can also produce deformation when an electric field is applied. Thus, the manufacturing process developed in this work is a preliminary step in the additive manufacturing of electrically activated DEAP actuators. In combination with the current work, this can provide a means for dual actuation mechanisms through DEAP actuation and pneumatic actuation along with the potential for DEAP embedded sensing. This will not only contribute to the state of the art in actuators with embedded sensors, but also allow for larger deformations, forces, and finer control in actuation. This dual functionality is particularly advantageous in applications where space and weight are constraints, allowing a single device to fulfill multiple roles. The development of such integrated systems could lead to significant advancements in fields such as robotics, where both sensing and actuation are critical.

## Data Availability

Data available upon request to corresponding author.
